# Similarities and differences between IL11 and IL11RA1 knockout mice for lung fibro-inflammation, fertility and craniosynostosis

**DOI:** 10.1038/s41598-021-93623-9

**Published:** 2021-07-08

**Authors:** Benjamin Ng, Anissa A. Widjaja, Sivakumar Viswanathan, Jinrui Dong, Sonia P. Chothani, Stella Lim, Shamini G. Shekeran, Jessie Tan, Narelle E. McGregor, Emma C. Walker, Natalie A. Sims, Sebastian Schafer, Stuart A. Cook

**Affiliations:** 1 Cardiovascular and Metabolic Disorders Program, Duke-National University of Singapore Medical School, Singapore, Singapore; 2 National Heart Research Institute Singapore, National Heart Centre Singapore, Singapore, Singapore; 3 Bone Biology and Disease Unit, St. Vincent’s Institute of Medical Research, Melbourne, Australia; 4 Department of Medicine, St. Vincent’s Hospital, The University of Melbourne, Melbourne, Australia; 5 MRC-London Institute of Medical Sciences, Hammersmith Hospital Campus, London, UK

**Keywords:** Extracellular signalling molecules, Interleukins

## Abstract

Loss of function (LOF) in *IL11RA* infers IL11 signaling as important for fertility, fibrosis, inflammation and incompletely penetrant craniosynostosis. The impact of LOF in *IL11* has not been characterized. We generated IL11 knockout (*Il11*^−/−^) mice that are born in expected ratios and have normal hematological profiles. Lung fibroblasts from *Il11*^−/−^ mice are resistant to pro-fibrotic stimulation with TGFβ1. Following bleomycin-induced lung injury, *Il11*^−/−^ mice are protected from pulmonary fibrosis and exhibit lesser ERK, STAT3 and NF-kB activation, reduced *Il1b*, *Timp1*, *Ccl2* and diminished IL6 expression, both at baseline and after injury: placing *Il11* activity upstream of *IL6* in this model. *Il11*^−/−^ female mice are infertile. Unlike *Il11ra1*^−/−^ mice, *Il11*^−/−^ mice do not have craniosynostosis, have normal long bone mass and reduced body weights. These data further establish the role of IL11 signaling in lung fibrosis while suggesting that bone development abnormalities can be associated with mutation of *IL11RA* but not *IL11*, which may have implications for therapeutic targeting of IL11 signaling.

## Introduction

Interleukin 11 (IL11) was originally described as a factor important for hematopoiesis, notably platelet production, but more recently found to drive fibro-inflammatory disorders^1^. IL11 is a member of the IL6 family of cytokines, which share the gp130 coreceptor, but while IL6 has been studied in very great detail with an armamentarium of genetic tools to dissect its function, IL11 remains poorly characterised^1, 2^.

It is apparent from the published literature that the majority of our understanding of the biology associated with loss-of-function (LOF) in IL11 signaling is inferred from studies of *IL11RA* mutant humans or mice^1^. The field of lL11 biology has lacked a mouse genetic model specific for IL11 LOF, which represents a gap in our understanding. This is important as, in the case of the family member IL6, there are both similarities and differences between effects of LOF in the *IL6* cytokine as compared to LOF in its cognate alpha chain receptor (*IL6RA*)^3, 4^. As such, it is possible that the phenotype of *IL11RA* LOF may not map precisely to IL11 function. Furthermore, studies of *IL11RA1* LOF have been conducted in a single mouse strain and there are additional genes close by in the targeted locus, which is a potential shortcoming.

Based on the genetic studies of *IL11RA* mutants, IL11 signaling is thought important for a number of phenotypes. *Il11ra1*-deleted female mice are infertile^5^ and mutation in *Il11ra1* in the mouse is associated with incompletely penetrant snout displacement and tooth abnormalities: a craniosynostosis-like phenotype^6^. Several human studies have identified individuals with *IL11RA* mutations who have mild features of craniosynostosis, joint laxity, scoliosis and delayed tooth eruption, although ascertainment bias has been suggested^6–8^. Unlike mice, female humans with mutations in *IL11RA* appear fertile in some studies^9^.

Here, we report the generation of mice with germline deletion of *Il11* that we characterise at baseline and in the context of pro-fibrotic stimulation in vitro and in vivo. We also report similarities and differences between the phenotypes of *Il11*^−/−^ and *Il11ra1*^−/−^ mice through direct experimentation and in comparison with the published *Il11ra1*^−*/*−^ literature (reviewed in Ref.^1^).

## Results

### Generation and anatomical characterization of *Il11* knockout mice

**Figure 1:**
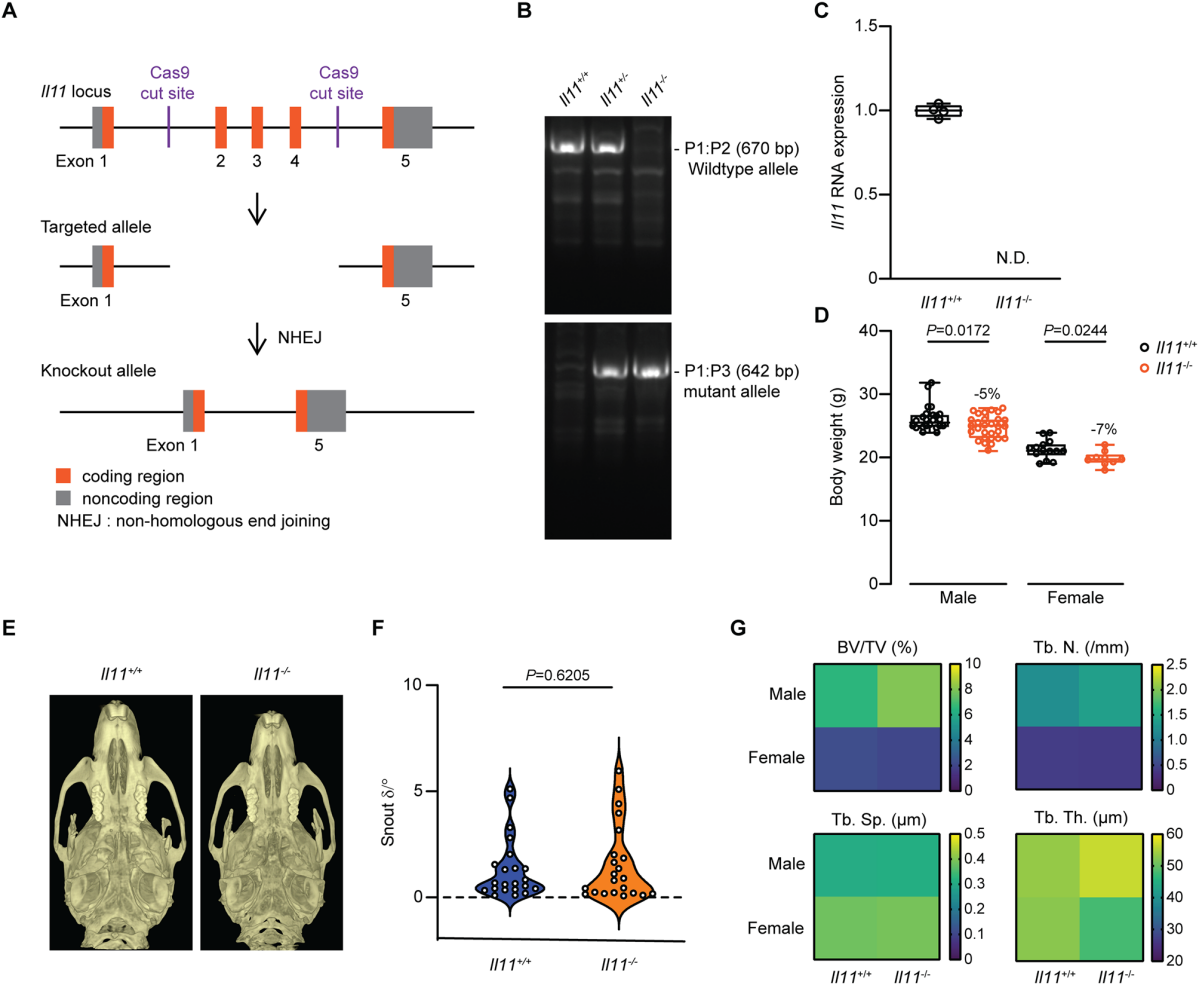
Generation and anatomical characterization of *Il11* knockout mice. (**A**) Schematic design of Crispr/Cas9 mediated deletion of exons 2 to 4 of the mouse *Il11* locus on a C57BL/6J background. (**B**) Representative genotyping of *Il11*-deficient or wild-type mice, showing a wild-type band (670 bp) and mutant band (642 bp). (**C**) RT-qPCR of *Il11* expression in whole lung tissue from wild-type and *Il11*^*−/−*^ mice (*n* = 4). N.D., not detected. (**D**) Body weight of 10–12 week old wild-type (male *n* = 26; female *n* = 14) and *Il11*^*−/−*^ mice (male *n* = 29; female *n* = 9). (**E**) Representative µCT images showing the ventral view of skulls from wild-type and *Il11*^*−/−*^ mice. (**F**) The degree of deviation from linear snout growth (δ/°) was determined in *Il11*^*−/−*^ as compared to wild-type mice (*Il11*^+*/*+^ *n* = *23* and *Il11*^*−/−*^* n* = 22). (**G**) Heatmaps showing the quantification of trabecular bone parameters of *Il11*^*−/−*^ and wild-type mice (12 weeks of age); n = 5–7 mice per genotype per gender and quantitative data are plotted in Supplementary Fig. S1. BV/TV, Trabecular bone volume per total volume, Tb.Th., trabecular thickness, Tb. N., trabecular number and Tb. Sp., trabecular separation. Data shown in C, D are: centre line, median value; box edges, 25th and 75th percentiles; whiskers, minimum and maximum values and data in F are shown as a violin plot. *P* values were determined by Student’s *t*-test.

Three separate transcripts of mouse *Il11* (ENSMUSG00000004371) have been annotated and using Crispr/Cas9, we deleted Exon 2–4 of the longest transcript (ENSMUST00000094892.11: Il11-201). This deletion causes a reading frame shift after the first two amino acids of IL11 resulting in a mutant 62 amino acids peptide that does not align to any known peptide sequences resulting in the inactivation of all known transcripts (Fig. 1A).

*Il11* knockout mice (*Il11*^−*/*−^) were generated on a C57BL/6J background and genotypes were determined by sequencing and PCR (Fig. 1B). To address whether the mutant alleles resulted in the loss of *Il11* RNA expression, we isolated total RNA from whole lung tissue from *Il11*^−*/*−^ mice and did not observe any detectable expression of *Il11* RNA by RT-qPCR in *Il11*^−/−^ mutants (Fig. 1C). We observed a slight (5–7% lower) but statistically significant reduction in body weights of male and female *Il11*^−*/*−^ mice (10–12 weeks old) as compared to age and gender matched wild-type controls (Fig. 1D), which has not been reported in *Il11ra1*^−*/*−^ mice of a similar age by us or others.

It is reported that approximately 40–50% of *Il11ra1*^−*/*−^ mice display a craniosynostosis-like phenotype^10^. We assessed the skulls of adult mice (> 12 weeks of age) in *Il11*^−*/*−^ mice (*n* = 22) and littermate control wild-type mice (*n* = 23) and compared data with an analysis of *Il11ra1*^−*/*−^ mice (*n* = 12) maintained locally, also on a C57BL/6J background. Similar to what was reported previously, we observed snout deformities in 42% of *Il11ra1*^−*/*−^ mice (5 out of 12 mice) (Supplementary Fig. S1A)^10^. However, we did not observe significant differences in the proportions of *Il11*^−*/*−^ mice with snout deformities as compared to littermate controls (*P* = 0.6) (Fig. 1E and Supplementary Fig. S1A). In separate blinded analyses by experts in the field using microcomputed tomography (µCT) analysis, it was confirmed that there was no significant difference in snout phenotypes in *Il11*^−*/*−^ mice as compared to wild-type mice, unlike snouts of *Il11ra1*^−*/*−^ mice that were distorted and served as a positive control (Fig. 1F and Supplementary Fig. S1B).

Previous studies of *Il11ra1*^−*/*−^ mice have shown that IL11RA1-deletion results in a high bone mass phenotype^11^. We characterized the bone phenotype of *Il11*^−*/*−^ mice by µCT analysis of the distal femora and showed that trabecular bone parameters were similar in *Il11*^−*/*−^ and wild-type mice (Fig. 1G and Supplementary Fig. S1C–F). In gross anatomy studies, the indexed organ-to-body weight ratios of the heart, lung, liver, kidney, spleen and pancreas were comparable in *Il11*^−*/*−^ and wild-type mice (Supplementary Fig. S2).

### Female *Il11* knockout mice are infertile

**Figure 2:**
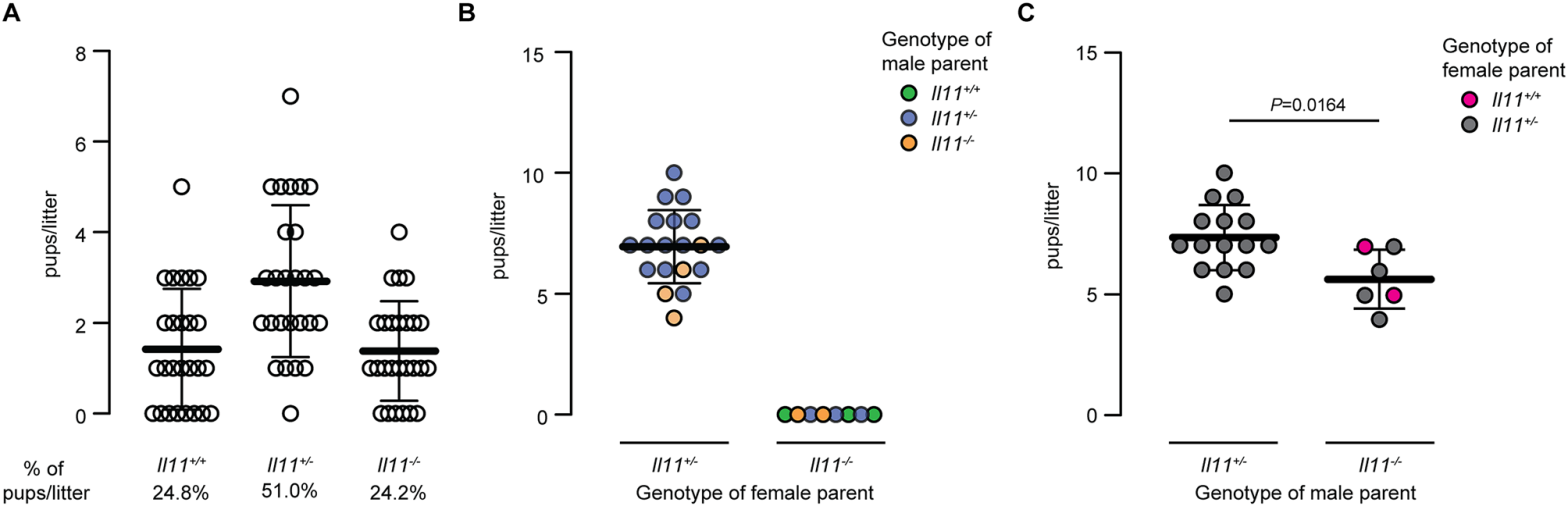
*Il11* knockout female mice are infertile. (**A**) Genotype distribution of pups per litter in heterozygous breeding (*Il11*^+*/−*^ X *Il11*^+*/−*^;* n* = 26 litters). (**B**) Litter size based on parents’ genotype. (**C**) Litter size based on the breeding of heterozygous *Il11*^+*/−*^ or homozygous *Il11*^*−/−*^ male mice with wild-type or heterozygous *Il11*^+*/−*^ female mice. Data shown as mean ± SD. *P* value was determined by Student’s *t* test.

Deletion of *Il11ra1* in mice leads to female infertility due to defective embryo implantation, whereas *Il11ra1*^−*/*−^ males are fertile^5, 12, 13^. We found that intercrosses of heterozygotes *Il11*^+*/*−^ mice gave rise to viable and apparently normal homozygous (*Il11*^−*/*−^) mutant mice in the expected Mendelian ratios (Fig. 2A). We determined whether maternal *Il11* expression is required for fertility by mating homozygous (*Il11*^−*/*−^) female mice with male mice of variable *Il11* genotype (*Il11*^+*/*+^, *Il11*^+*/*−^ or *Il11*^−*/*−^) and found that female mice deficient for *Il11* never had a detectable pregnancy nor gave birth to offspring (Fig. 2B), which mirrors the infertility phenotype of homozygous *Il11ra1*^−*/*−^ female mice^5^. Crossing homozygous (*Il11*^−*/*−^) male mice with either wild-type (*Il11*^+*/*+^) or heterozygous (*Il11*^+*/*−^) female mice resulted in viable offspring of expected Mendelian ratios. However, litter sizes derived from *Il11*^−*/*−^ male mice were significantly smaller as compared to intercrosses of heterozygotes (Fig. 2C).

### Blood hematology and chemistry profiles are normal in *Il11* knockout mice

We evaluated the hematological profile of adult *Il11*^−*/*−^ mice (10–14 weeks of age) and observed that null mice had normal peripheral red and white blood cell counts as well as normal platelet counts and volumes as compared to wild-type mice (Table 1). Likewise, we profiled serum chemistry and observed normal levels of serum markers of liver function (albumin, alanine aminotransferase, total bilirubin), kidney function (blood urea nitrogen, sodium and potassium) and bone turnover (alkaline phosphatase, calcium and phosphate) in *Il11*^−*/*−^ mice (Table 1). These data indicate that *Il11*^−*/*−^ mice have ordinary blood hematological and chemistry profiles under normal physiological conditions.

**Table 1:** Hematology and serum metabolic profiles of *Il11* knockout mice.

Blood counts	*Il11*^+*/*+^ (*n* = 5)	*Il11*^*−/−*^ (*n* = 7)	Sig.^a^
WBC (10^9^/L)	6.74 ± 2.60	6.30 ± 1.60	NS
Lymphocytes (10^9^/L)	5.00 ± 1.83	4.84 ± 1.67	NS
Monocytes (10^9^/L)	0.23 ± 0.16	0.31 ± 0.31	NS
Neutrophils (10^9^/L)	1.52 ± 1.65	1.15 ± 0.98	NS
RBC (10^12^/L)	10.18 ± 2.46	9.02 ± 0.21	NS
HGB (g/dL)	11.98 ± 2.23	13.52 ± 0.43	NS
HCT (%)	45.42 ± 12.16	40.66 ± 1.16	NS
Platelets (10^9^/L)	521 ± 358	387 ± 75	NS
Mean Platelet Volume (fl)	6.12 ± 0.16	6.15 ± 0.26	NS

Serum metabolic markers	*Il11*^+*/*+^ (*n* = 5)	*Il11*^*−/−*^ (*n* = 6)	Sig.^a^
Albumin (g/dL)	3.34 ± 0.32	3.23 ± 0.30	NS
Alkaline Phosphatase (U/L)	89.20 ± 7.33	98.83 ± 21.15	NS
Alanine Aminotransferase (U/L)	35.00 ± 20.10	59.17 ± 27.69	NS
Amylase (U/L)	730 ± 142	696 ± 91	NS
Total Bilirubin (mg/dL)	0.32 ± 0.04	0.32 ± 0.04	NS
Blood Urea Nitrogen (mg/dL)	20.00 ± 5.39	19.67 ± 3.56	NS
Calcium (mg/dL)	9.62 ± 0.16	9.67 ± 0.55	NS
Phosphate (mg/dL)	10.88 ± 2.20	10.57 ± 0.96	NS
Glucose (mg/dL)	328 ± 108	389 ± 91.91	NS
Sodium (mmol/L)	147 ± 1.87	143 ± 3.92	NS
Potassium (mmol/L)	5.24 ± 0.44	5.43 ± 0.67	NS
Total Protein (g/dL)	4.34 ± 0.17	4.10 ± 0.13	NS
Globulin (g/dL)	0.98 ± 0.19	0.83 ± 0.23	NS

Data shown as mean ± SD.

*WBC* white blood cells, *RBC* red blood cells, *HGB* hemoglobin count, *HCT* hematocrit, *NS* non-significant.

^a^Significance was determined using Bonferroni post-hoc test.

### IL11 is required for myofibroblast differentiation

**Figure 3:**
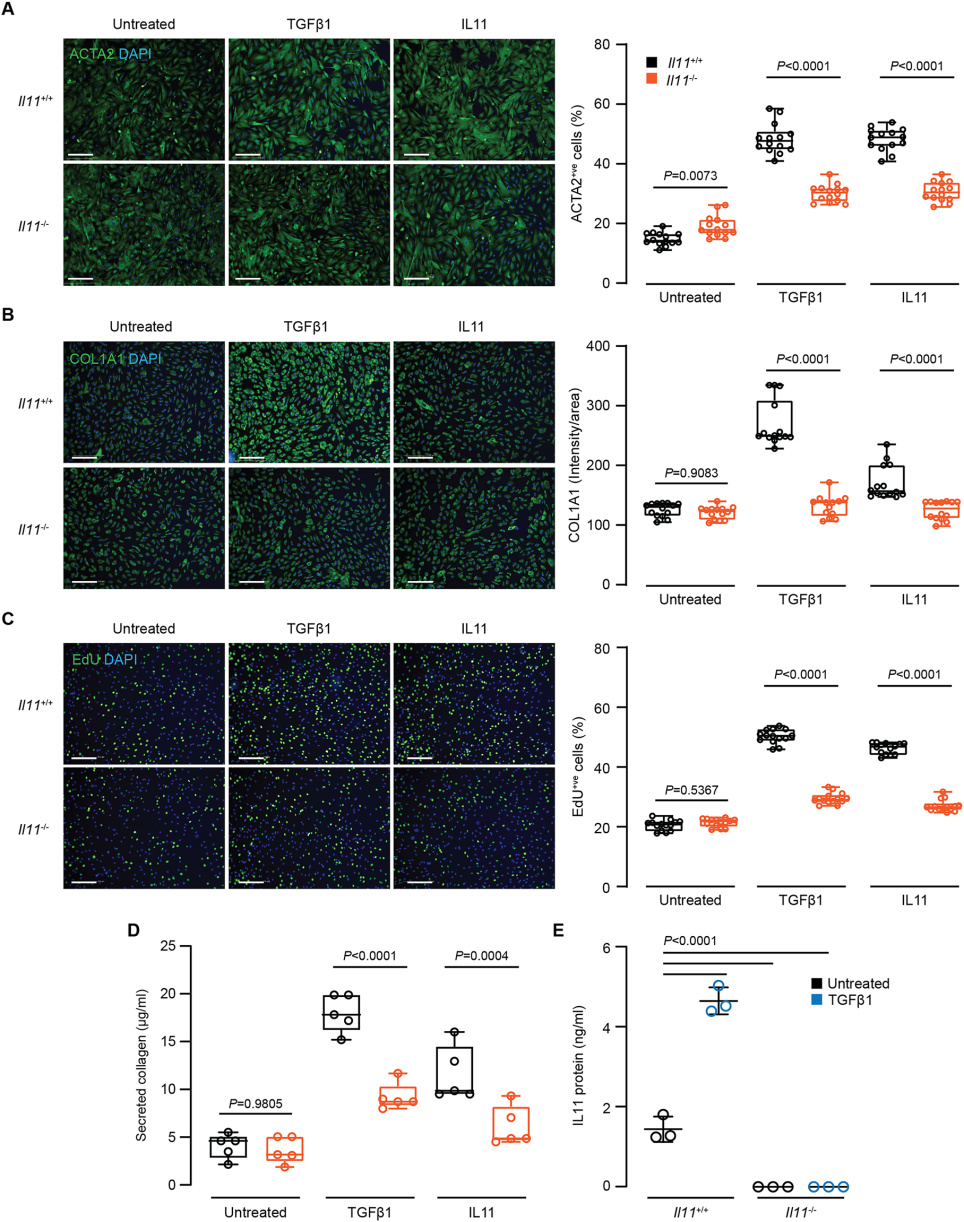
Reduced activation of primary lung fibroblasts from *Il11* knockout mice. Automated immunofluorescence quantification of (**A**) ACTA2^+ve^ cells, (**B**) COL1A1 expression (intensity/area) and (**C**) EdU^+ve^ cells in TGFβ1- or IL11-treated lung fibroblasts from *Il11*^*−/−*^ or wild-type mice (5 ng/ml, 24 h). One representative dataset from two independent biological experiments is shown (14 measurements per condition per experiment). Scale bars: 200 µm. (**D**) Secreted collagen concentrations in supernatant of cells treated as described in (**A**,**B**) were quantified (*n* = 5). (**E**) Secreted IL11 in culture supernatant from TGFβ1-treated lung fibroblasts from *Il11*^*−/−*^ or wild-type mice (5 ng/ml, 24 h; *n* = 3). Data in (**A**–**D**) are shown as: centre line, median value; box edges, 25th and 75th percentiles; whiskers, minimum and maximum values; and shown as mean ± SD in (**E**). *P* values in (**A**–**D**) were determined by ANOVA (Sidak’s test) and by ANOVA (Tukey’s test) in (**E**).

We found previously that TGFβ1-induced myofibroblast transdifferentiation is impaired in *Il11ra1*^−*/*−^ lung fibroblasts^14^. To examine whether the loss of the endogenous IL11 autocrine feed-forward loop similarly perturbed fibroblast activation, we stimulated lung fibroblasts from *Il11*^−*/*−^ mice with recombinant mouse TGFβ1 or IL11 (5 ng/ml; 24 h) and monitored fibroblast activation using automated high-throughput immunofluorescence imaging and Sirius red-based quantification of secreted collagen. In keeping with the data from *Il11ra1*-deleted fibroblasts, the differentiation of *Il11*^−*/*−^ fibroblasts into ACTA2^+ve^ and COL1A1 expressing myofibroblasts following TGFβ1 stimulation was significantly diminished (Fig. 3A,B). Cell proliferation (as determined by EdU^+ve^ staining) and secreted collagen levels into the culture supernatant were also significantly reduced in *Il11*^−*/*−^ fibroblasts following TGFβ1 stimulation (Fig. 3C,D).

We next addressed whether disruption of the IL11 locus prevented IL11 protein expression by performing ELISA on culture supernatants and found that IL11 protein was not expressed by *Il11*^−*/*−^ lung fibroblasts at baseline or after TGFβ1 stimulation (Fig. 3E). Interestingly, recombinant mouse IL11 (5 ng/ml) did not fully restore pro-fibrotic phenotypes in *Il11*^−*/*−^ fibroblasts (Fig. 3A–D). We determined the expression of IL11RA in *Il11*^−*/*−^ lung fibroblasts by immunofluorescence staining and detected reduced levels of IL11RA expression in IL11-deleted cells (Supplementary Fig. S3), which might contribute towards a diminished response to exogenous IL11 stimulation, as may the absence of an IL11 feed-forward autocrine loop.

### Bleomycin-induced pulmonary fibrosis is attenuated in *Il11* knockout mice

**Figure 4:**
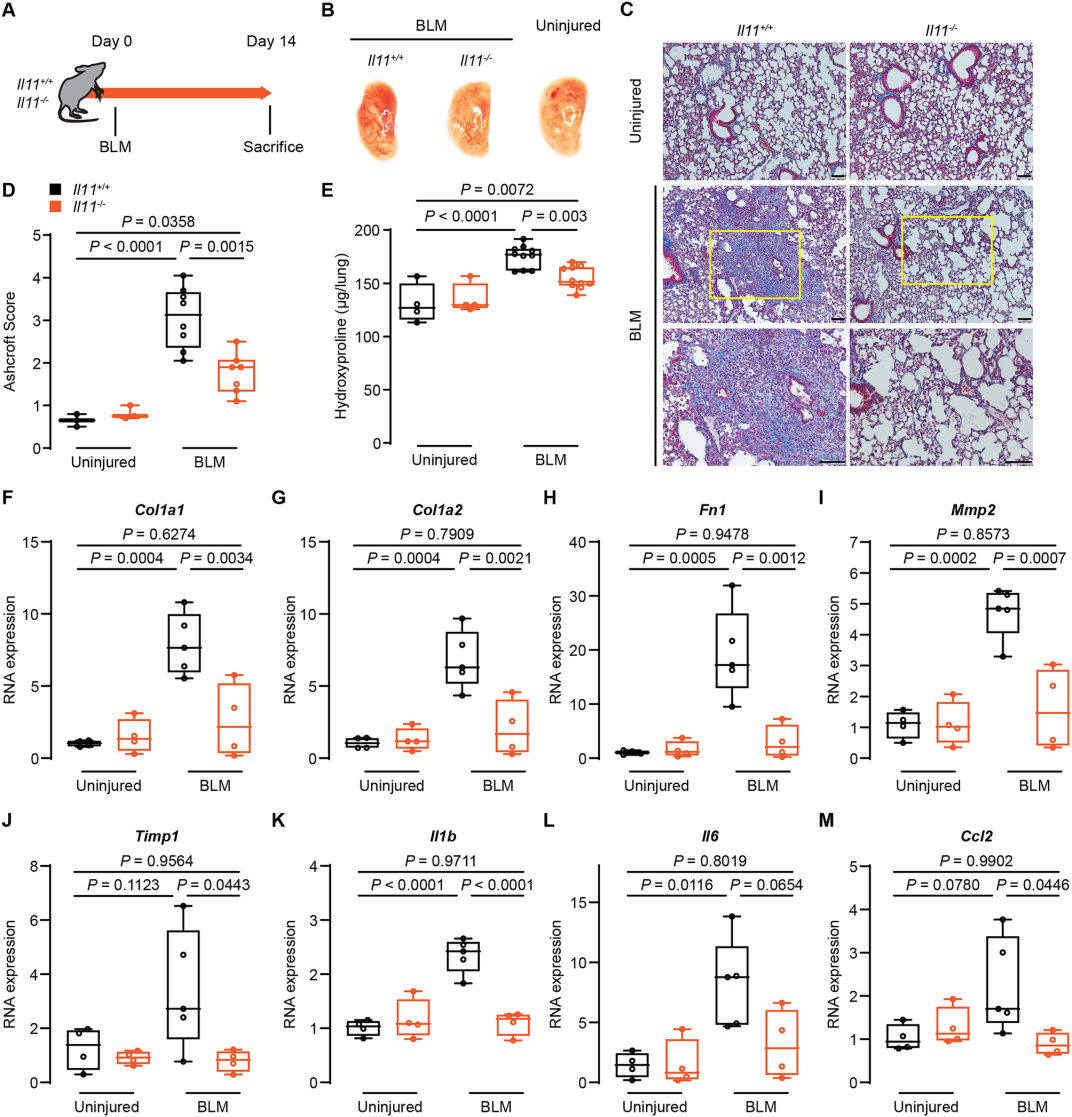
Bleomycin-induced pulmonary fibrosis is attenuated in *Il11* knockout mice. (**A**) Schematic showing the induction of lung fibrosis in *Il11*^*−/−*^ and wild-type mice. A single dose of bleomycin (BLM) was administered oropharyngeally and the mice were sacrificed 14 days post-BLM. (**B**) Representative gross lung anatomy of *Il11*^*−/−*^ and wild-type mice 14 days post-BLM. (**C**) Masson’s trichrome staining of lung sections, (**D**) histology assessment of fibrosis and (**E**) total lung hydroxyproline content of *Il11*^*−/−*^ and wild-type mice 14 days post-BLM. Scale bars: 100 µm. (*Il11*^+*/*+^ uninjured n = 3–4; *Il11*^+*/*+^  + BLM n = 7–10; *Il11*^*−/−*^ uninjued n = 3–4; *Il11*^*−/−*^ + BLM n = 7–10). Relative RNA expression of (**F**) *Col1a1*, (**G**) *Col1a2*, (**H**) *Fn1*, (**I**) *Mmp2*, (**J**) *Timp1*, (**K**) *Il1b*, (**L**) *Il6* and (**M**) *Ccl2* in lung lysates from *Il11*^*−/−*^ and wild-type mice 14 days post-BLM (*n* = 4). Data shown as: centre line, median value; box edges, 25th and 75th percentiles; whiskers, minimum and maximum values. *P* values were determined by ANOVA (Tukey’s test).

IL11 expression is elevated in the mouse lung after bleomycin (BLM)-induced injury and BLM-induced lung fibrosis is attenuated in *Il11ra1*^−*/*−^ mice^15^. To determine if the genetic deletion of *Il11* provided similar protection, we challenged *Il11*^−*/*−^ mice with BLM. In preliminary experiments, we found that *Il11*^−*/*−^ mice had a higher mortality rate compared to *Il11ra1*^−*/*−^ mice, with the same dose/protocol we used previously^15^. We therefore lowered the dose of BLM and chose to evaluate the mice lungs at an earlier time point (14 days post-BLM) when fibrosis is equally established (Fig. 4A).

By gross morphology analysis, we observed reduced macroscopic lung damage in *Il11*^−*/*−^ mice than that seen in wild-type mice (Fig. 4B). As compared to wild-type mice, following BLM injury *Il11*^−*/*−^ mice gained more weight (Day 0–14), had reduced pulmonary infiltrates and alveolar septal thickening whereas indexed lung weights were unchanged (Supplementary Fig. S4). Consistent with these effects, blinded histological analysis of Masson’s trichrome stained lung sections showed that *Il11*^−*/*−^ mice had reduced parenchymal disruption and fibrosis (Fig. 4C,D). These changes were associated with significantly lower total lung hydroxyproline (collagen) content in *Il11*^−*/*−^ mice (Fig. 4E).

Evaluation of fibrotic gene expression in lung lysates in a subset of representative lung samples showed reduced RNA levels of extracellular matrix and protease genes such as *Col1a1*, *Col1a2*, *Fn1*, *Mmp2* and *Timp1* in BLM-challenged *Il11*^−*/*−^ mice as compared to wild-type mice (Fig. 4F–J). Reduced expression of several inflammatory response genes (such as *Il1b*, *Il6* and *Ccl2*) were also seen in the lungs of *Il11*^−*/*−^ mice following BLM injury (Fig. 4K–M).

**Figure 5:**
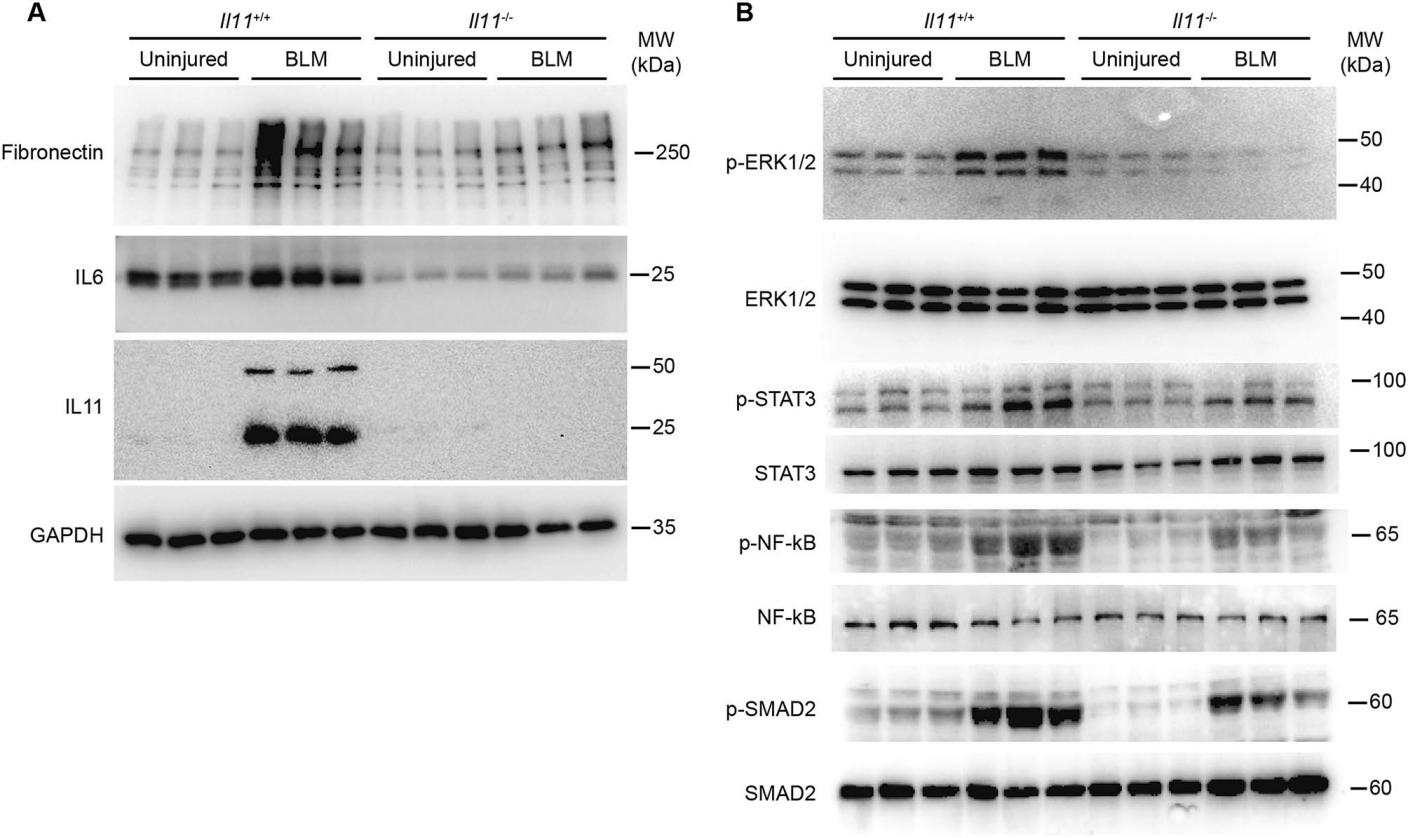
Bleomycin-induced pulmonary fibronectin, IL6, IL11 expression and ERK, STAT3, NF-kB and SMAD2 phosphorylation in *Il11* knockout and wild type mice. Western blots of (**A**) fibronectin, IL6 and IL11 and (**B**) phosphorylated and total protein levels ERK1/2, STAT3, NF-kB and SMAD2 in lung homogenates of *Il11*^*−/−*^ and wild-type mice 14 days post-BLM (*n* = 3 for each genotype/condition).

Western blot analysis in a subset of representative lung samples showed that IL11 protein expression was strongly upregulated in the lungs of BLM-injured wild-type mice and was not expressed at all in the lungs of *Il11*^−*/*−^ mice at all, as expected (Fig. 5A). Furthermore, in BLM-treated *Il11*^−*/*−^ mice, lung protection was accompanied by reduced pulmonary fibronectin and IL6 protein expression (Fig. 5A) and reduced activation of multiple signaling pathways implicated in lung fibro-inflammation including ERK, STAT3, NF-kB and SMAD2 (Fig. 5B). Notably, IL6 levels in *Il11*^−*/*−^ mice were lower than wild type control levels even at baseline, in the absence of lung injury. These data show that *Il11*^−*/*−^ mice are protected from BLM-induced lung fibrosis and inflammation, similar to *Il11ra1*^−*/*−^ mice^15, 16^.

## Discussion

Here we provide a phenotypic description of mice with germline deletion of *Il11* and explore how this relates to the phenotypes of *Il11ra1*-null mice, published previously. We show that IL11 signaling in *Il11*^−*/*−^ mice is important for qualitative fibrotic phenotypes in fibroblasts in vitro and lung fibrosis in vivo. These data, taken together with recent studies of *Il11ra1*^−*/*−^ mice and the use of anti-IL11 or anti-IL11RA antibodies in mouse models of fibrosis^14, 15, 17^, firmly establish IL11-mediated ERK signaling as of central importance for fibrosis.

*Il11ra1*^−*/*−^ mice have normal hematopoiesis at baseline and after bone marrow or hemolytic stress^12^ and long term administration of neutralizing antibodies against IL11 or IL11RA have no effect on blood counts in mice^17^. In agreement with mouse data, there is no description of hematological abnormalities in patients with *IL11RA1* mutations^18^. While IL11 is still considered by some to have a role in platelet homeostasis, the data shown here in *Il11*^−/−^ mice provide a further line of evidence that IL11 is unrelated to physiological hematopoiesis, although we did not study bone marrow stress.

It was surprising to observe differences in skull morphology and bone phenotypes between *Il11*^−*/*−^ and that published, and replicated by us, for *Il11ra1*^−*/*−^ genotypes, both maintained on C57BL/6J backgrounds. This said, it was recently shown that *Ccl27* expression may be disrupted at the *Il11ra1* locus in *Il11ra1*^−*/*−^ mice^19^ although this is unrelated to the craniosynostosis phenotypes seen in humans with bi-allelic *IL11RA* LOF mutations. The fact that *Il11*^−*/*−^ mice do not have snout deformities implies that this is unrelated to the loss of IL11 signaling per se. In a recent study, a biallelic non-synonymous variant in *IL6ST* (gp130), which results in a loss of IL11-signaling, conferred a craniosynostosis phenotype, which replicated in a mouse model, although again this phenotype is mediated at the membrane receptor level^20^. However, female mutant mice in the study were fertile and also exhibited reduced LIF signaling. Intriguingly, while LOF mutations in *IL11* are common in the general population they have not been associated with craniosynostosis despite large scale sequencing projects^8^ whereas *IL11RA* mutations are widely reported^6, 7^.

IL11 signaling has been reported to play a role in skeletal remodeling by promoting the differentiation and function of osteoblasts and osteoclasts^21–23^. Intriguingly, *Il11*^−*/*−^ mice did not exhibit a high bone mass phenotype, or other bone abnormalities, as observed previously in *Il11ra1*^−*/*−^ mice^11^. This suggests further that the LOF in *IL11* itself does not affect bone homeostasis and provides a safety signal if long-term anti-IL11 therapy was ever considered as a therapeutic intervention. While adult *Il11*^−*/*−^ mice were found to have lower body weights than their littermate controls, this was not apparent in adult *Il11ra1*^−*/*−^ mice which had comparable body weights to their corresponding wild-type controls. This was not evaluated further here and requires additional study.

There are parallels between the variant phenotypes between *Il11ra1-* and *Il11-*deficient mice and those seen for *Il6*- and *Il6ra*-deficient mice. For instance, physiologically important immune phenotypes associated with loss of canonical IL6-mediated JAK/STAT signaling are seen with both *Il6*- or *Il6ra* genotypes. However, dissimilar ERK-mediated wound healing phenotypes are only seen in *Il6ra* mutants, which are dominant over *Il6* LOF effects (i.e. IL6-independent)^4^. Furthermore, *Il6* or *Il6ra* mutant mice also have different responses to experimental models of colitis^3^.

A possible explanation for the difference between IL6 family member alpha chain receptor versus ligand mutants could relate to reduced/no interaction of mutant alpha chains with the gp130. Thus, biallelic LOF in one alpha chain (e.g. IL6R) might reduce its competition for the shared gp130 receptor and potentiate the activity of another gp130-binding alpha chain (e.g. IL11RA). This premise could account for the increased ERK signaling seen in IL6RA mutant mice, perhaps reflecting increased IL11-driven ERK signaling^4^. This concept is also suggested further by human genetics: autosomal recessive mutations in gp130 are associated with craniosynostosis, whereas dominant negative (autosomal dominant) variation is not^24, 25^.

There is variation in reported fertility phenotypes associated with LOF mutations in the IL11 pathway in mice and humans (Table 2). Female mice lacking *Il11RA1* are infertile due to defective decidualization in response to embryo implantation^5, 13^. In contrast, women with homozygous *IL11RA* variants appear able to reproduce^9^, which could be explained by species differences in decidualization^26^. In this study, we found that *Il11*^−*/*−^ female mice are infertile, consistent with a recent report^27^. Interestingly, we observed a reduction in litter sizes from *Il11*^−*/*−^ male mice, suggesting additional male-related effects of IL11 on fertility, which warrants further study.

**Table 2:** Fertility reported with genetic loss-of-function in IL11 signaling in mice and humans.

Gene	Mutation/variant	Gender effects	References
**Human**			
*IL11RA*	Exon 4 donor splice site (c.479 + 6T > G)	Females: fertile	^9^
**Mouse**			
*Il6st*	Gp130 p.R279Q (selective IL11 signaling deficiency)	Females: homozygous mutants have reduced litter size Males: homozygous mutants have reduced litter size	^20^
*Il11ra1*	Deletion of Exons 8–13	Females: homozygous mutants are infertile due to defective decidulization Males: normal fertility	^5, 12^
*Il11ra1*	Hypomorphic mutations	Females: fertility of homozygous mutants are severely impaired Males: normal fertility	^13^
*Il11*	Crispr/Cas9 frameshift in Exon 3	Females: homozygous mutants are infertile (data not shown) Males: not reported	^27^
*Il11*	Crispr/Cas9 deletion of Exons 2–4. Frameshift from 2nd amino acid (aa), mutant 62 aa protein	Females: homozygous mutants are infertile Males: homozygous mutants have reduced litter size	This study

While IL11 is increasingly recognized as important for tissue fibrosis, more recent data has shown a role for IL11 signaling in inflammatory fibroblasts and stromal immunity in the lung and colon^16, 28, 29^. Fitting with this, we found that *Il11*^−*/*−^ mice were protected from BLM-induced lung inflammation with lower *Il1b*, *Il6* and *ccl2* mRNA levels, diminished immune cell NF-kB and STAT3 activation and reduced pulmonary infiltrates. Notably, at the protein level, IL6 was not only not upregulated in the injured lung of *Il11*^−*/*−^ mice and IL6 levels were also lower in *Il11*^−*/*−^ mouse lungs at baseline. We point out that, for reasons outlined above, we do not attempt to compare the magnitude of protection against lung damage following BLM challenge between the *Il11*^−*/*−^ mice used here and previous data from *Il11ra1*^−*/*−^ mice^15^. Instead, we wish to emphasize the shared qualitative protection against lung fibro-inflammation between *Il11*^−*/*−^ and *Il11ra1*^−*/*−^ genotypes.

In conclusion, loss of IL11 signaling due to mutation in either *Il11* or *Il11RA1* is protective against lung fibrosis that relates to reduced autocrine IL11 activity in myofibroblasts^14, 30^. However, the craniosynostosis and bone phenotypes seen in *Il11RA1* deficient mice are not seen in *Il11*^−*/*−^ mice, suggesting that developmental, STAT3-related bone effects are not due to defective IL11 signaling per se and instead, specific to *Il11RA1* LOF. This is in agreement with a recent study that, apart from the fertility issue we describe here, reported no other phenotype in *Il11*^−*/*−^ mice^27^. This is important, as therapeutic targeting of IL11 signaling for fibrotic lung and liver diseases is being considered and it may be that targeting the ligand has potential advantages over the receptor. To facilitate further study of IL11, the *Il11*^−*/*−^ mice described in this manuscript have been made available to the scientific community at the Jackson Laboratories repository.

## Materials and methods

### Animal studies

All experimental procedures were approved and conducted in accordance with guidelines set by the Institutional Animal Care and Use Committee at SingHealth (Singapore) and the SingHealth Institutional Biosafety Committee and with the recommendations in the *Guidelines on the Care and Use of Animals for Scientific Purposes of the National Advisory Committee for Laboratory Animal Research* (NACLAR) and with the *Animal Research: Reporting of In Vivo Experiments* (ARRIVE) guidelines. Animals were maintained in a specific pathogen-free environment and given ad libitum access to food and water.

#### Generation of Il11 knockout mice

Crispr/Cas9 technique was used to knock out the IL11 gene (ENSMUST00000094892.11: Il11-201 transcript). Specific single guide RNA (sgRNA) sequences with recognition sites on introns 1 and 4 along with Cas9 were microinjected into fertilized C57BL/6J zygotes, and subsequently transferred into pseudopregnant mice (Shanghai Model Organisms, Centre, Inc). It is predicted that this deletion would cause a shift in the reading frame from the splicing of coding sequences present within exons 1 and 5, resulting in the generation of a mutant peptide (62 amino acids in length) that does not align to that of known proteins. This effectively results in the inactivation of the gene. Successfully generated F0 mice were identified by PCR and sequencing and further backcrossed to wild type C57BL/6J mice and maintained on this background. Wild-type allele was identified by a 670 bp PCR product using genotyping primer sequences as follows: P1: 5′-CGGGGGCGGACGGGAGACG-3′ and P2: 5′-CCAGGAGGGATCGGGTTAGGAGAA-3′. Whereas, mutant (knockout) alleles were identified by a second PCR reaction using primers P1 and P3: 5′-CAGCTAGGGACGACACTTGAGAT-3′.

#### Bleomycin model of lung injury

The bleomycin model of lung fibrosis was performed as previously described^15^. Briefly, female mice (8–10 weeks of age) were anesthetized by isoflurane inhalation and subsequently administered a single dose of bleomycin (Sigma-Aldrich) oropharyngeally at 0.5 mg/kg body weight in a volume of saline not exceeding 50 µl per mouse. Uninjured animals received equal volumes of saline as sham controls. Mice were sacrificed 14 days post-bleomycin administration and lungs were collected for downstream analysis.

### Analysis of craniosynostosis-like snout phenotype and trabecular bone mass analysis

*Il11ra1*^*−/−*^ mice on a C57BL/6J background were originally described in Ref.^12^ and obtained from The Jackson’s laboratory. For the analysis of skull phenotypes of *Il11*^*−/−*^, *Il11ra1*^*−/−*^ and wild-type mice, the heads of both male and female adult mice (> 12 weeks of age) were dissected and cleaned by removing the soft tissue surrounding the skull. The bones were fixed in 10% neutral buffered formalin. For the analysis of trabecular bone mass, the hindlimbs of 12-week old mice were dissected and tibiae and femora bones were fixed in 10% neutral buffered formalin. Micro-computed tomography of the skulls and femora of *l11*^*−/−*^, *Il11ra1*^*−/−*^ and wild-type mice was performed using the Skyscan 1276 system and analysed using NRecon (version 1.6.9.8), Dataviewer (version 1.4.4) and CT analyzer (version 1.11.8.0) as previously described^31^. For the analysis of skull phenotypes, classification was performed by scoring for the presence (craniosynostosis-like phenotype) or absence (no phenotype) of twisted snouts by two independent investigators blinded to genotypes, and concordant scores were obtained between the investigators. Deviation from linear nasal bone growth was determined by assessing the angle between the tip of the snout bone and the sagittal suture at the base of the frontal bone by ImageJ software analysis (v1.8)^32^.

### Hematologic analysis

Blood was collected by cardiac puncture from anesthetized male and female adult mice (10–14 weeks of age). Differential red and white cell counts, hematocrit, hemoglobin and platelet counts were determined using the VetScan HM5 hematology analyzer (Abraxis Inc.). Blood chemistry profiles from male and female mice were determined using the VetScan VS2 system, partnered with the VetScan comprehensive diagnostic profile discs (Abraxis Inc.).

### Reagents

Recombinant proteins: Mouse TGFβ1 (7666-MB, R&D Systems), mouse IL11 (rmIL11, UniProtKB: P47873, GenScript). Antibodies: anti-smooth muscle actin (ab7817, abcam), anti-Collagen I (ab34710, abcam), anti-IL11RA (ab125015, abcam), goat anti-mouse Alexa Flour 488-conjugated secondary antibody (ab150113, abcam), goat anti-rabbit Alexa Flour 488-conjugated secondary antibody (ab150077, abcam), DAPI (D1306, Thermo Fisher Scientific). Primary antibodies for Western blots include: anti-Fibronectin antibody (ab2413, Abcam); anti-IL6 (12912, Cell Signaling); anti-p-ERK1/2 (4370, Cell Signaling), anti-ERK1/2s (4695, Cell Signaling), anti-p-SMAD2 (ab216482, Abcam), anti-p-NF-kB (ab28856, Abcam), anti-p-STAT3 (4113, Cell Signaling), anti-SMAD2 (5339, Cell Signaling), anti-NF-kB (8242, Cell Signaling), anti-STAT3 (4904, Cell Signaling) and anti-GAPDH (2118, Cell Signaling). Monoclonal anti-IL11 antibody (X203), generated in our previous study^15^, was used to detect IL11 protein expression in tissue lysates. Secondary antibodies for Western blots include: anti-rabbit HRP (7074, Cell Signaling) or anti-mouse HRP (7076, Cell Signaling).

### Primary mouse lung fibroblasts cultures

Primary mouse lung fibroblast were isolated from 8 to 12 weeks old *Il11*^*−/−*^ and wild-type mice as previously described^15^. Tissues were minced, digested for 30 min with mild agitation at 37 °C in DMEM (11995-065, Gibco) containing 1% penicillin/streptomycin (P/S, 15140-122, Gibco) and 0.14 Wunsch U ml^−1^ Liberase (5401119001, Roche). Cells were subsequently cultured in complete DMEM supplemented with 10% fetal bovine serum (10500, Hyclone), 1% P/S, in a humidified atmosphere at 37 °C and 5% CO_2_. Fresh medium was renewed every 2–3 days. Fibroblasts were allowed to explant from the digested tissues and enriched via negative selection with magnetic beads against mouse CD45 (leukocytes), CD31 (endothelial) and CD326 (epithelial) using a QuadroMACS separator (Miltenyi Biotec) according to the manufacturer’s protocol. All experiments were carried out at low cell passage (< P3) and cells were cultured in serum-free media for 16 h prior to stimulation.

### Operetta high-content imaging and analysis

Immunofluorescence imaging and quantification of fibroblast activation were performed on the Operetta High Content Imaging System (PerkinElmer) as previously described^14, 15^. Briefly, lung fibroblasts were seeded in 96-well CellCarrier black plates (PerkinElmer) and following experimental conditions, the cells were fixed in 4% paraformaldehyde (Thermo Fisher Scientific) and permeabilized with 0.1% Triton X-100 in phosphate-buffered saline (PBS). EdU-Alexa Fluor 488 was incorporated using Click-iT EdU Labelling kit (C10350, Thermo Fisher Scientific) according to manufacturer’s protocol. The cells were then incubated with primary antibodies (anti-ACTA2 or anti-COL1A1) and visualized using anti-mouse or anti-rabbit Alexa Flour 488-conjugated secondary antibodies. Plates were scanned and images were collected with the Operetta high-content imaging system (PerkinElmer). Each treatment condition was run in duplicate wells, and 14 fixed fields were imaged and analysed per condition. The percentage of activated myofibroblasts (ACTA2^+ve^ cells) and proliferating cells (EdU^+ve^ cells) was quantified using the Harmony software version 3.5.2 (PerkinElmer). Quantification of COL1A1 immunostaining was performed using the Columbus software (version 2.7.2, PerkinElmer), and fluorescence intensity was normalized to cell area.

### Lung histology analysis

Freshly dissected left lungs from each *Il11*^*−/−*^ and wild-type mouse in the bleomycin experiment were fixed in 10% formalin overnight, dehydrated and embedded in paraffin and sectioned for Haematoxylin and Eosin or Masson’s trichrome staining as described previously^15^. Histological analysis for fibrosis was performed blinded to genotype and treatment exposure according to Ashcroft scoring method^33^.

### Quantification of collagen content in culture supernatant and lung tissue

Detection of soluble collagen in the supernatant of lung fibroblasts cultures was performed as previously described^15^. Briefly, the cell culture supernatant was concentrated using a Polyethylene glycol concentrating solution (90626, Chondrex) and collagen content was quantified using a Sirius red collagen detection kit (9062, Chondrex), according to the manufacturer’s protocol. Total lung hydroxyproline content of the right caudal lobes of each mouse mouse in the bleomycin experiment were was measured as previously described^15^, using the Quickzyme Total Collagen assay kit (Quickzyme Biosciences).

### ELISA

Detection of secreted IL11 into the supernatant of lung fibroblast cultures was performed using the mouse IL-11 DuoSet ELISA kit according to manufacturers’ instructions.

### RT-qPCR

Total RNA was extracted from snap-frozen mouse right lung tissues using Trizol reagent (Invitrogen) followed by RNeasy column (Qiagen) purification and cDNA was prepared using an iScript cDNA synthesis kit (Biorad) following manufacturer's instructions. Quantitative RT–PCR gene expression analysis was performed with QuantiFast SYBR Green PCR kit (Qiagen) using a StepOnePlus (Applied Biosystem). Relative expression data were normalized to *Gapdh* mRNA expression using the 2^−ΔΔCt^ method. Primers sequences used for are as follows: *Il11* 5′-AATTCCCAGCTGACGGAGATCACA-3′ and 5′-TCTACTCGAAGCCTTGTCAGCACA-3′; *Col1a1* 5′-GGGGCAAGACAGTCATCGAA-3′ and 5′-GTCCGAATTCCTGGTCTGGG-3′; *Col1a2* 5′-AGGATTGGTCAGAGCAGTGT-3′ and 5′-TCCACAACAGGTGTCAGGGT-3′; *Fn1* 5′-CACCCGTGAAGAATGAAGA-3′ and 5′-GGCAGGAGATTTGTTAGGA-3′; *Mmp2* 5′-ACAAGTGGTCCGCGTAAAGT-3′ and 5′-AAACAAGGCTTCATGGGGGC-3′; *Timp1* 5′-GGGCTAAATTCATGGGTTCC-3′ and 5′-CTGGGACTTGTGGGCATATC-3′; *Ccl2* 5′-GAAGGAATGGGTCCAGACAT-3′ and 5′-ACGGGTCAACTTCACATTCA-3′; *Il6* 5′-CTCTGGGAAATCGTGGAAAT-3′ and 5′-CCAGTTTGGTAGCATCCATC-3′; Il1b 5′-CACAGCAGCACATCAACAAG-3′ and 5′-GTGCTCATGTCCTCATCCTG-3′; *Gapdh* 5′-CTGGAAAGCTGTGGCGTGAT-3 and 5′-GACGGACACATTGGGGGTAG-3′.

### Western blot analysis

Total proteins were extracted from snap-frozen mouse right lung tissues using RIPA lysis buffer (Thermo Fisher Scientific) and separated by SDS-PAGE, transferred to a PVDF membrane (Biorad), and incubated overnight with the appropriate primary antibodies. Blots were visualized using the ECL detection system (Pierce) with the appropriate secondary antibodies.

### Statistical analysis

All statistical analyses were performed using Graphpad Prism (version 8). Statistical analyses were performed using two-sided Student’s t-test, or ANOVA as indicated in the figure legends. For comparisons between multiple treatment groups, *P* values were corrected for multiple hypothesis testing using Sidak’s test, Tukey’s test or Bonferonni’s post-hoc test. *P* values < 0.05 were considered statistically significant.

## Supplementary Information

Supplementary Information.

## Data Availability

All data generated and analysed in the current study are presented in the manuscript or available from the corresponding author upon request.
