# Supplementary material for: Similarities and differences between IL11 and IL11RA1 knockout mice for lung fibro-inflammation, fertility and craniosynostosis
Source: Sci Rep. 2021 Jul 8;11:14088. doi: 10.1038/s41598-021-93623-9 (PMC8266813; doi:10.1038/s41598-021-93623-9)

Supplementary Figures:

Similarities and differences between IL11 and IL11RA1 knockout mice for lung fibro-inflammation, fertility and craniosynostosis

Benjamin Ng[1,2†], Anissa A. Widjaja[1†], Sivakumar Viswanathan[1], Jinrui Dong[1], Sonia P. Chothani[1], Stella Lim[1], Shamini G. Shekeran[1], Jessie Tan[1,2], Narelle E. McGregor[3], Emma C. Walker[3], Natalie A. Sims[3,4], Sebastian Schafer[1,2], Stuart A. Cook[1,2,5,6*]

*Corresponding author
Stuart A. Cook
Email: stuart.cook@duke-nus.edu.sg

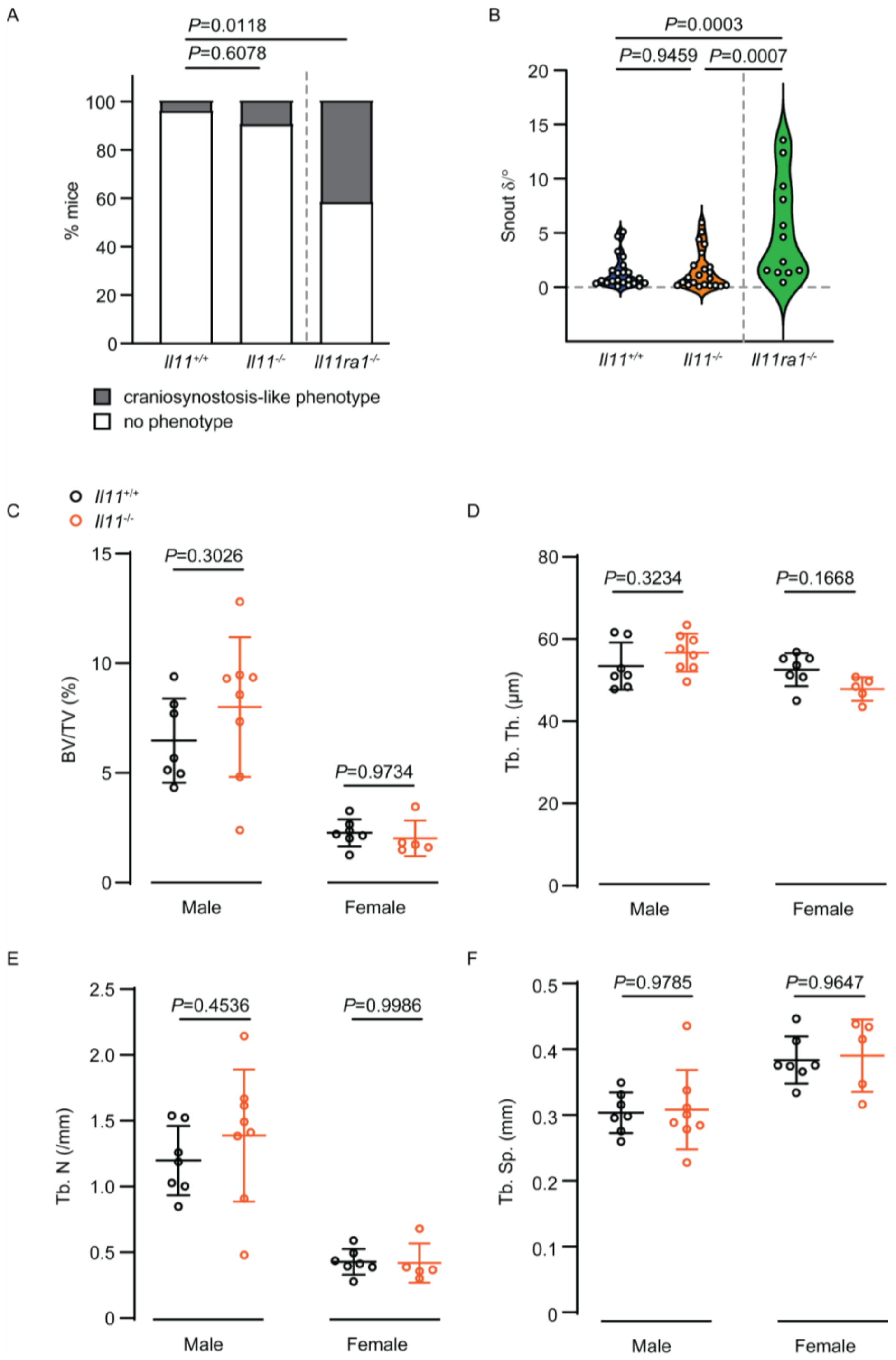

Supplementary Figure S1. Skull and trabecular bone parameters of adult *Il11* knockout mice. (**A**) Proportion of mice with craniosynostosis-like phenotype and (**B**) the degree of deviation from linear snout growth (δ/˚) was determined in *Il11*$^{-/-}$ and *Il11ra1*$^{-/-}$ as compared to *Il11*$^{+/+}$ mice (*Il11*$^{+/+}$ *n=23, Il11*$^{-/-}$ *n*=22, *Il11ra1*$^{-/-}$ *n*=12), all on a C57BL/6J background. Data points in panel B for *Il11*$^{+/+}$ and *I11*$^{-/-}$ mice are presented in main Fig. 1F and replotted here as a reference. (**C-F**) The µCT analysis of the distal femora of male and female *Il11*$^{-/-}$ and *Il11*$^{+/+}$ mice (12 weeks of age); n=5-7 mice per genotype per gender. (A) Trabecular bone volume per total volume (BV/TV; %), (B) trabecular thickness (Tb.Th.), (C) trabecular number (Tb. N) and (D) trabecular separation (Tb. Sp.). Data in A are shown as stacked bar graphs; B shown as violin plot; C-F are shown as mean ± SD; *P* values were determined by Fisher's exact test in panel A, ANOVA (Tukey's test) in panel B and by Student's *t*-test in panels C-F.

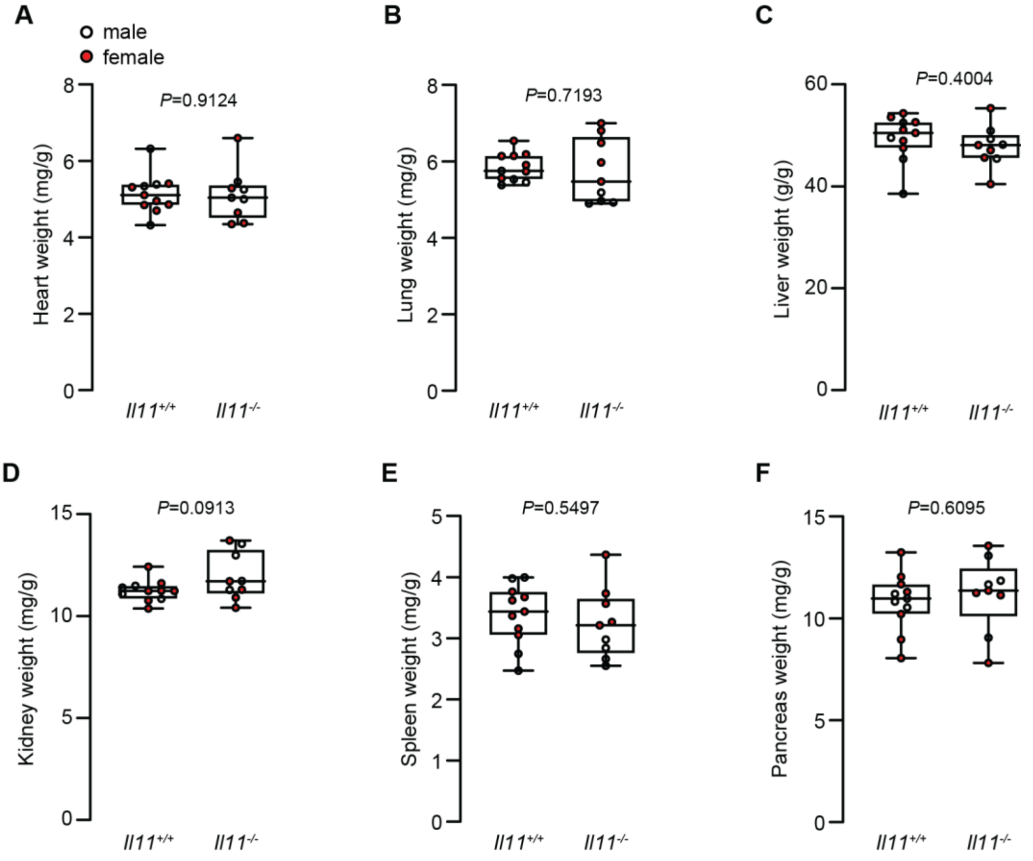

Supplementary Figure S2. Anatomical characterization of adult *Il11* knockout mice. (**A**) Heart weight-, (**B**) lung weight-, (**C**) liver weight-, (**D**) kidney weight-, (**E**) spleen weight- and (**F**) pancreas weight-to-body weight indices of male and female *Il11*$^{-/-}$ and wild-type mice (10-14 weeks of age) (n=9-10 per genotype). Data shown as: centre line, median value; box edges, 25th and 75th percentiles; whiskers, minimum and maximum values; *P* values were determined by Student's *t*-test

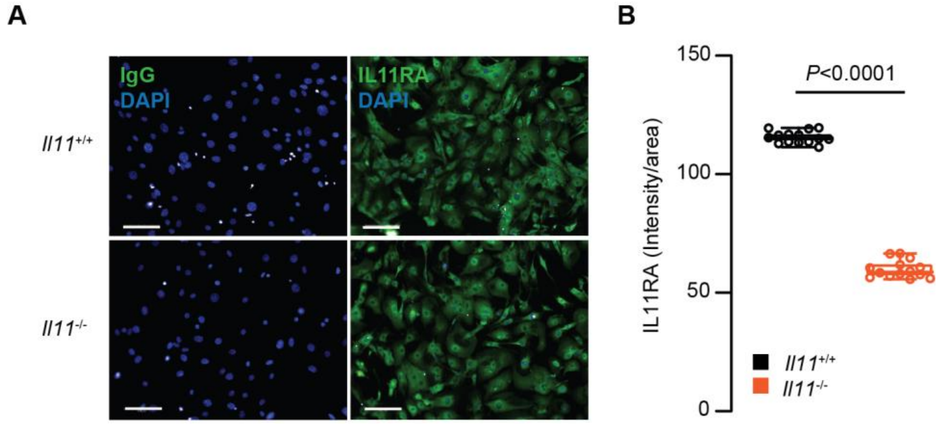

Supplementary Figure S3. Fibroblasts from *Il11* knockout mice have reduced IL11RA expression. (A) Representative images and (B) quantification of IL11RA immunostaining in *Il11*$^{-/-}$ and wild type fibroblasts. Scale bars: 200 µm. Data shown as mean ± SD; *P* value was determined by Student's *t*-test.

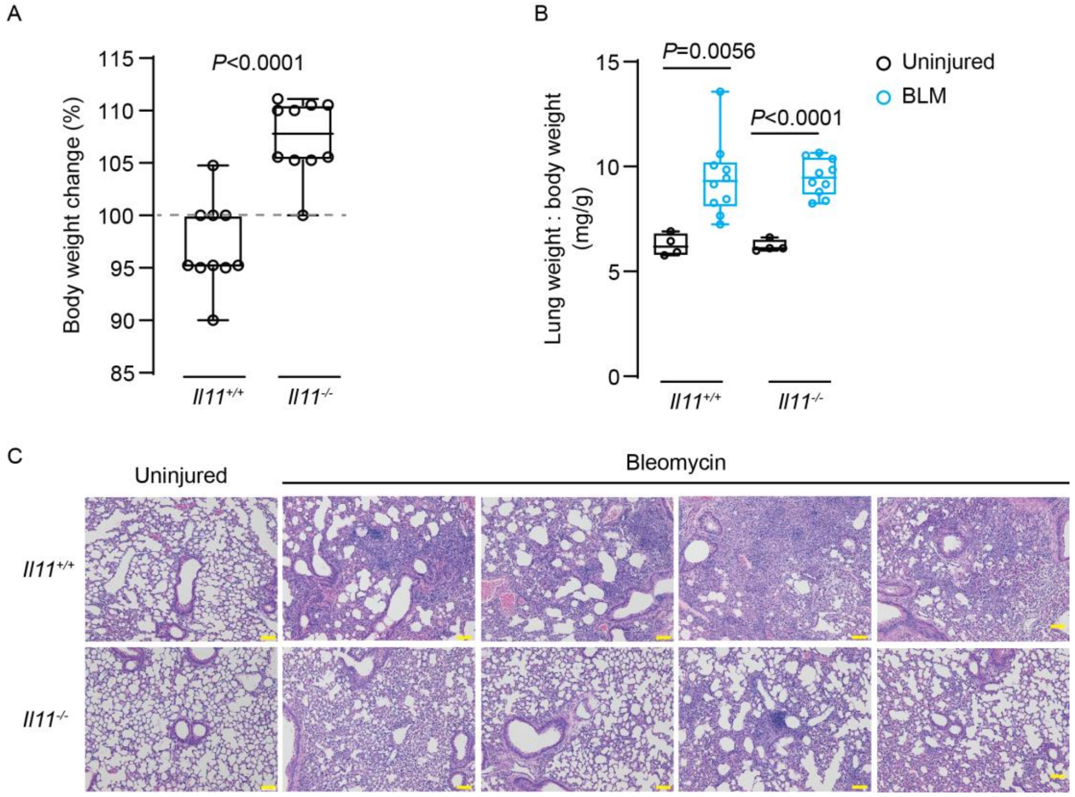

Supplementary Figure S4. Body and lung weights of *Il11* knockout and wild-type mice post-bleomycin injury. (**A**) Percentage body weight change post-BLM challenge (day 14 versus day 0), (**B**) lung weight to body weight index and (**C**) hematoxylin and eosin staining of lung sections from *Il11*$^{-/-}$ and wild-type mice 14 days post-BLM. Scale bars: 100 µm. Data shown as: centre line, median value; box edges, 25th and 75th percentiles; whiskers, minimum and maximum values; *P* values were determined by Student's *t*-test.

Uncropped blots for Fig 5A

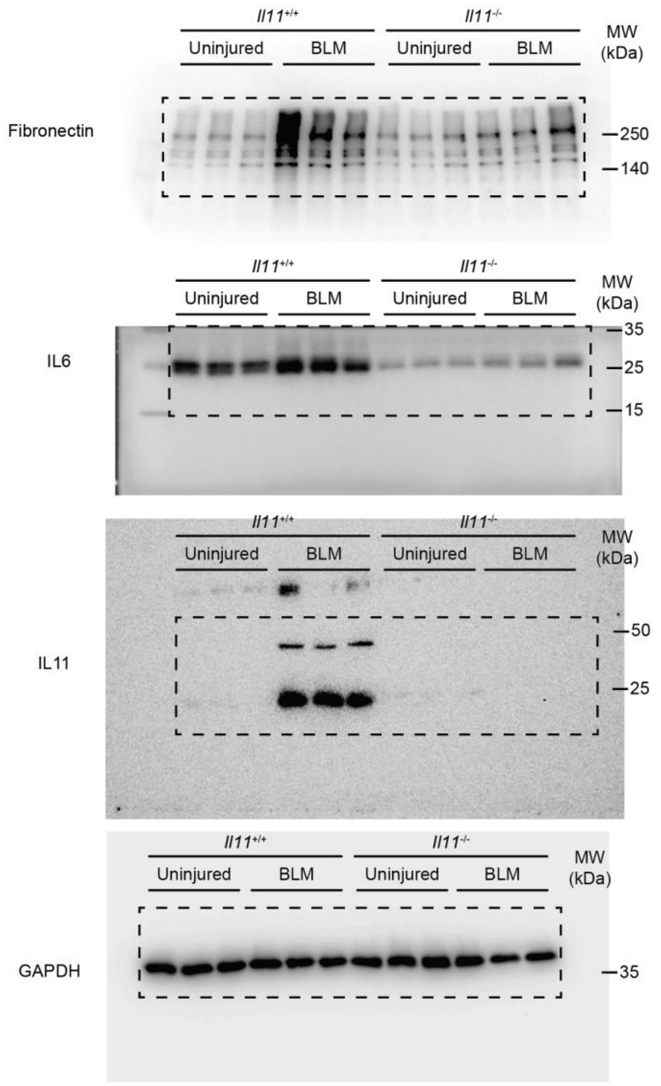

Uncropped blots for Fig 5B

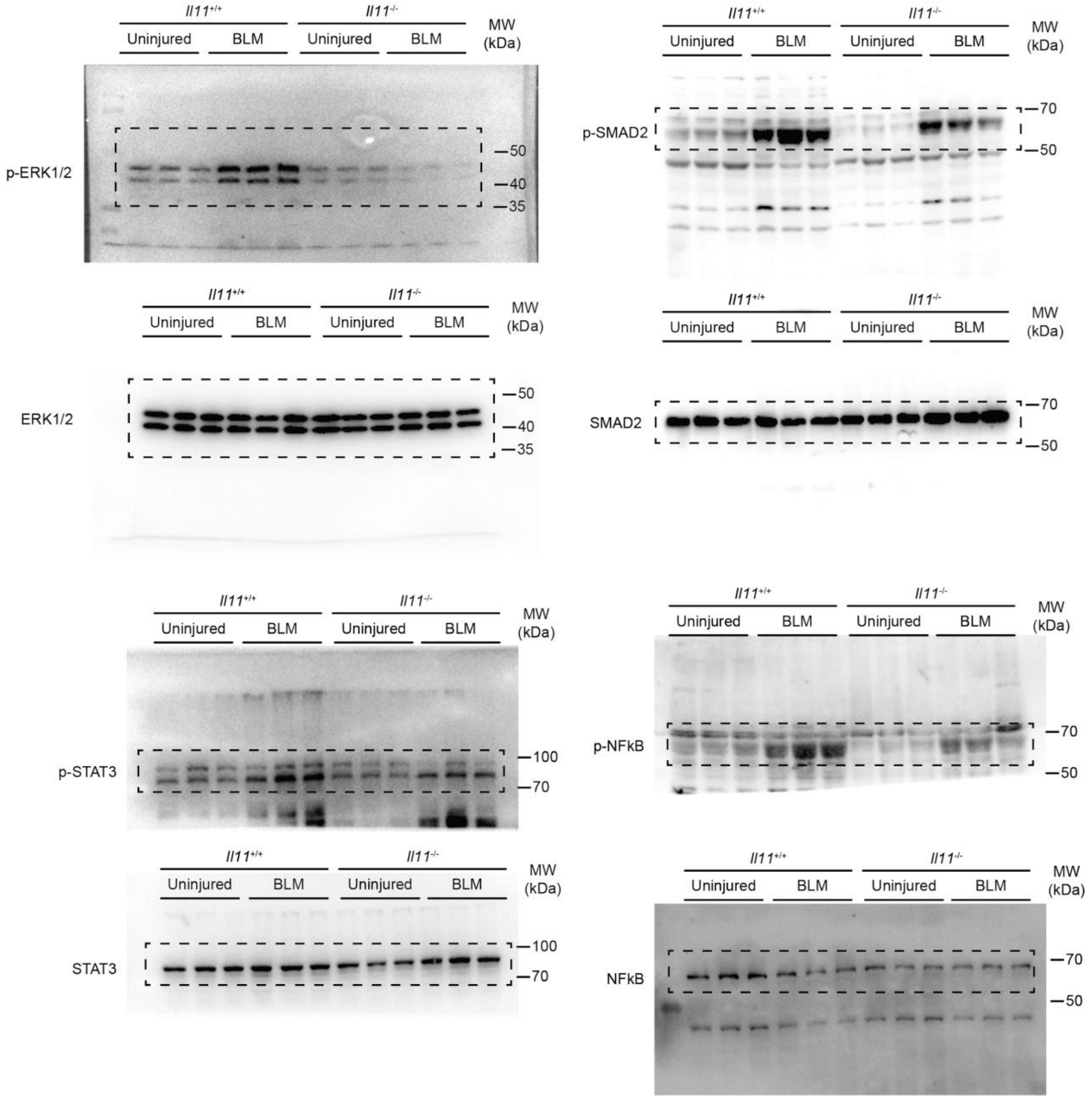

Supplement: Supplementary file 1 — Supplementary Information. [file 41598_2021_93623_MOESM1_ESM.pdf]
